# Assessing the effect of containment measures on the spatio-temporal dynamic of COVID-19 in Italy

**DOI:** 10.1007/s11071-020-05853-7

**Published:** 2020-08-08

**Authors:** Maria Michela Dickson, Giuseppe Espa, Diego Giuliani, Flavio Santi, Lucia Savadori

**Affiliations:** 1 Department of Economics and Management, University of Trento, Via Inama 5, 38122 Trento, TN Italy; 2 Department of Economics, University of Verona, Verona, VR Italy

**Keywords:** COVID-19, Italy, Spatio-temporal model, Spatial dependence, Quarantine

## Abstract

This paper aims at investigating empirically whether and to what extent the containment measures adopted in Italy had an impact in reducing the diffusion of the COVID-19 disease across provinces. For this purpose, we extend the multivariate time-series model for infection counts proposed in Paul and Held (Stat Med 30(10):118–1136, 2011) by augmenting the model specification with B-spline regressors in order to account for complex nonlinear spatio-temporal dynamics in the propagation of the disease. The results of the model estimated on the time series of the number of infections for the Italian provinces show that the containment measures, despite being globally effective in reducing both the spread of contagion and its self-sustaining dynamics, have had nonlinear impacts across provinces. The impact has been relatively stronger in the northern local areas, where the disease occurred earlier and with a greater incidence. This evidence may be explained by the shared popular belief that the contagion was not a close-to-home problem but rather restricted to a few distant northern areas, which, in turn, might have led individuals to adhere less strictly to containment measures and lockdown rules.

## Introduction

At the time of writing, the COVID-19 pandemic represents a worldwide emergency. After the first pneumonia cases due to the SARS-CoV-2 virus were diagnosed at the end of December 2019 in the Chinese city of Wuhan [25], the disease spread in many countries at various speeds and with different effects. As of 20 April 2020, the USA was the most affected country in the world in terms of absolute number of contagions, followed by some European countries, such as Spain, Italy and France, which occupied the top three positions. Considering the different populations of these areas, Italy maintains the World ominous world record for the highest in case fatality rate (13.3%), defined as the proportion of deaths from the disease compared to the total number of people diagnosed with that disease over a certain period of time. Although these numbers are still uncertain due to under-reported infections and will be liable to adjustment [16], since the first infection was detected on 20 February 2020 in the province of Lodi (Lombardy region, northern Italy), the epidemic appeared to be very aggressive in this country. Within a few days, the disease spread throughout all Italian provinces, exhibiting nonlinear dynamics in all the areas.

Although all of the mechanisms behind the diffusion of COVID-19 in Italy are not currently completely clear and need to be further investigated by the scientific community, it was immediately evident that some measures to prevent the diffusion needed to be adopted. Though at the beginning of the outbreak, only ten towns in Lombardy and one in the Veneto region had been quarantined (the so-called *red area*), on 8 March 2020, a reinforced containment area (RCA), which had the aim of avoiding movements of people entering and leaving the territories, was established for the entire territory of the Lombardy region and 14 other northern provinces (five in Emilia-Romagna, five in Piedmont, three in Veneto and one in the Marche region). The removal of limits in the previous internal smaller red area meant that the infection began to flow throughout the RCAs, probably creating a proximity contagion and an amplification effect, such as the one that occurs in contaminated hospitals when a virus bounces back and forth within a restricted area. In addition, since the establishment of the RCA was leaked in the mass media before its entry into force, people were moved to flee en masse in an attempt to leave the region and return to their home towns. This created a predictable and preventable second wave of contagions that went from the north to the south of the peninsula because the northern area represents the industrial, economic and financial core of the country, and many people commute there to work. Some regional governors immediately ordered the quarantine of people who came from the RCA, but few attempts were made in that phase to check that this was actually done. As a result of these adverse effects of containment measures applied sparsely over the territory, on 11 March 2020, the Italian government issued a DPCM decree which made Italy a protected area, extending the quarantine to the whole country.

In addition to all this, during the outbreak of disease diffusion, ambivalent messages were shared in the mass media by Italian politicians and institutions to describe the new influenza, ranging from a situation under total control (a disease similar to seasonal flu) to war images depicting the military being used to enforce the quarantine in the original outbreak epicentre. Discrepancies were observed, especially at local level, due to different actual applications of containment measures by regions, provinces and municipalities, in addition to heterogeneous protocols in hospitalisation and in swabbing suspected cases. These contrasting and divergent communications were the origin of great confusion, especially because a virus is an “unobservable” risk [23], and the mechanisms of defence are based on the observation of others’ behaviour [18]. These features certainly contributed to the waste of precious time in the containment of the epidemic, and it is not surprising that people reacted chaotically to the various steps that led to the country-wide lockdown of Italy, nor is it surprising that the epidemic led to nonlinear contagious rates in different geographical areas.

In light of the above, there is no doubt that in order to study the outbreak of COVID-19, it is essential to consider both the temporal and spatial components of its diffusion [9]. Due to the strong local differences in the effects of the disease and to the different moments chosen to impose lockdowns throughout Italy, there is now great interest in studying the effect of lockdown on the diffusion of the disease. To this end, we extend the multivariate time-series model for infection counts proposed in Paul and Held [19]. In particular, following the approach suggested in Altan and Karasu [2] and Altan et al. [3] of combining traditional time-series models with modern statistical learning methods, we augment its specification with B-spline regressors to account for the complex nonlinear spatio-temporal dynamics that may have characterised the spread of COVID-19 infections in Italy. This methodology allows to assess whether there have been local differences in the effects of restrictions.

The paper is structured as follows. In Sect. 2, the adopted endemic–epidemic time-series mixed-effects generalised linear model for areal disease counts is presented and accordingly extended and adapted to study COVID-19. Section 3 presents the results of the model estimated using the Italian data at the province level for the period 24 February–20 April 2020. In Sect. 4, the results are discussed. Finally, the last section offers concluding remarks and indicates directions for future developments in this field.

## Methodological framework

### Model specification

The number of SARS-CoV-2 infections detected in the Italian provinces is a phenomenon with various features which, from a statistical point of view, should be taken into account.

The first is time dependence. Since the daily number of recorded infections is the result of a contagion mechanism which, from a modelling point of view, is well described through a branching process (see, for example, [8, 12]), time dependence necessarily arises. Moreover, the incubation period of COVID-19, which [15] estimated to last between 2.2 days (2.5th percentile) and 11.5 days (97.5th percentile), further contributes to the temporal dependence in the time series of the observed number of daily infections.

The second feature to be considered is the spatial dependence which arises amongst neighbouring provinces. This form of dependence originates from the transmission of SARS-CoV-2 throughout the Italian provinces because of movements of people, mainly for business reasons. The small geographical size of most Italian provinces and the high population density of several provinces in northern Italy leads to the high mobility of Italians between the provinces where they live and other neighbouring provinces, thus facilitating the spread of the virus throughout the territory.

The third important feature, which is relevant from a modelling point of view, is the marked heterogeneity amongst Italian provinces from geographical, economic and administrative points of view. Differences in terms of population density and in the structure of the economy between the northern and southern regions, as well as between urban and rural provinces, directly affect the mobility of the population across provinces and thus determine the intensity of the spatial dependence already mentioned. On the other hand, the partial autonomy of regional governments in managing the health emergency, as well as the regionally based organisation of the public health system, contributed to an inhomogeneous capability and differing political resolutions in dealing promptly with the epidemic. From a statistical point of view, these issues, if relevant, would entail that both the degree of time dependence in the number of daily infections and the exposure to the risk of an epidemic (which would result in an increase/decrease in the number of daily infections) vary from province to province.

These features motivated the adoption of the model proposed in Held et al. [13] and Paul and Held [19], namely a spatio-temporal generalised linear mixed effect model for count data, which has been successfully applied to predict the spread of infectious diseases in several studies (see, for example, [1, 4]). In the following, the structure of the model and the way it has been implemented to describe the number of COVID-19 infections in the Italian provinces are illustrated and discussed.

Let $t=0,1,\dots ,56$ be the time index of days between 24 February and 20 April 2020 (the time frame considered in this paper—see Sect. 3.1) and $r=1,2,\dots ,107$ be the index of Italian provinces. The number of infections observed on day *t* in province *r* is denoted by $Y_{r,t}$ and modelled as a negative binomial distribution conditionally to past observed values, that is:

$$\begin{aligned} Y_{r,t}\vert Y_{r,t-1},Y_{r,t-2},\ldots \sim \text {NegBin}(\mu _{r,t},\, \psi ), \end{aligned}$$

$\mu _{r,t}=\mathbb {E}(Y_{r,t}\vert Y_{r,t-1},Y_{r,t-2},\ldots )$ being the conditional mean of $Y_{r,t}$, and $\psi \ge 0$ being the overdispersion parameter which makes the conditional variance of $Y_{r,t}$ equal to $\mu _{r,t}\,(1+\psi \,\mu _{r,t})$. Note that if $\psi =0$, the conditional distribution of $Y_{r,t}$ degenerates to the Poisson distribution.

The main equation of the conditional expected number of contagions $\mu _{r,t}$ is the following:

1 $$\begin{aligned} \mu _{r,t} =\nu _{r,t}+\lambda _{r,t}\,Y_{r,t-2} +\phi _{r,t}\sum _{h\ne r}w_{r,h}\,Y_{h,t-1}. \end{aligned}$$

The three terms on the right-hand side of Eq. (1) correspond to the three components of the model: the endemic, epidemic-within and epidemic-between. From a terminological point of view, terms *epidemic* and *endemic*, when referring to the components of the model, have been inherited from Paul and Held [19], although terms *within* and *between* introduced in Giuliani et al. [9] are adopted in this paper in order to distinguish between the temporal and spatial terms which Paul and Held [19] jointly refer to as the *epidemic component*. In this regard, it is worth pointing out that the term *endemic* originates from the role that the component plays in the model, and it does not imply any epidemiological qualification of COVID-19 in the population of the Italian provinces.

The endemic component ($\nu _{r,t}$) is modelled by means of a log-linear equation consisting of three elements. Firstly, there is a province-specific random effect on the intercept which accounts for heterogeneous exposure of provinces to the initial risk of contagion. Secondly, resident population is included as a regressor, so that differences in size amongst provinces are accounted for. Thirdly, a basis of five B-splines of the fourth degree is included by means of four regressors in order to model the temporal trend shared by all Italian provinces in the number of contagions.

**Fig. 1:**
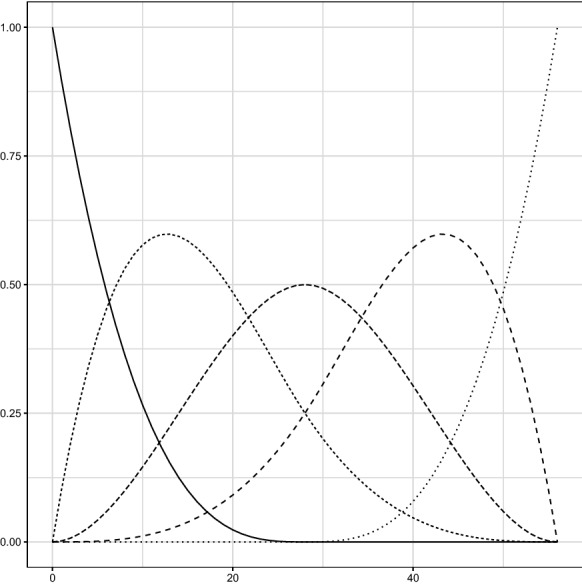
The basis of five B-splines of the fourth degree over the interval [0, 56], which corresponds to the time frame considered in the paper (24 February–20 April 2020)

The basis of B-splines (depicted in Fig. 1) is a basis of functions which enables the evolution of the endemic component (as well as the other parameters of the model, as illustrated below) to be modelled semi-parametrically. This permits the possible effects of quarantine measures to be caught without defining any dummy variables which would inevitably affect the estimates and could bias the interpretation of the results. For an illustration and a discussion of the basis of B-splines functions in functional data analysis, see [22].

The final equation of the endemic component $\nu _{r,t}$ is thus:

2 $$\begin{aligned} \ln (\nu _{r,t})=\alpha _r^{(\nu )}+\gamma ^{(\nu )}\,\ln (\text {rpop}_{r}) +\sum _{j=1}^4\beta ^{(\nu )}_jB_{j,t}^{(\nu )}, \end{aligned}$$

where $\alpha _r^{(\nu )}\sim \mathcal {N}(\alpha ^{(\nu )},\sigma _\nu ^2)$ is the random intercept; $\text {rpop}_{r}$ is the relative resident population of province *r*, computed as the ratio between the resident population of province *r* and the average resident population of the Italian provinces, and finally, $B_{j,t}^{(\nu )}$ is the value of the *j*th B-spline of the basis computed at time *t*.

Three clarifications are needed. Firstly, Eq. (2) includes the relative resident population of province *r* solely for numerical reasons: the model changes only in the average value of the intercept $\alpha ^{(\nu )}$ if the population of province *r* is included instead. Secondly, only four out of five B-splines are included in Eq. (2) because of the presence of the intercept $\alpha ^{(\nu )}$, which completes the basis: if the full basis of B-splines is included, perfect collinearity amongst regressors would emerge. Finally, the functional basis of B-splines is defined over the range of days between 24 February 2020 and 20 April 2020.

**Fig. 2:**
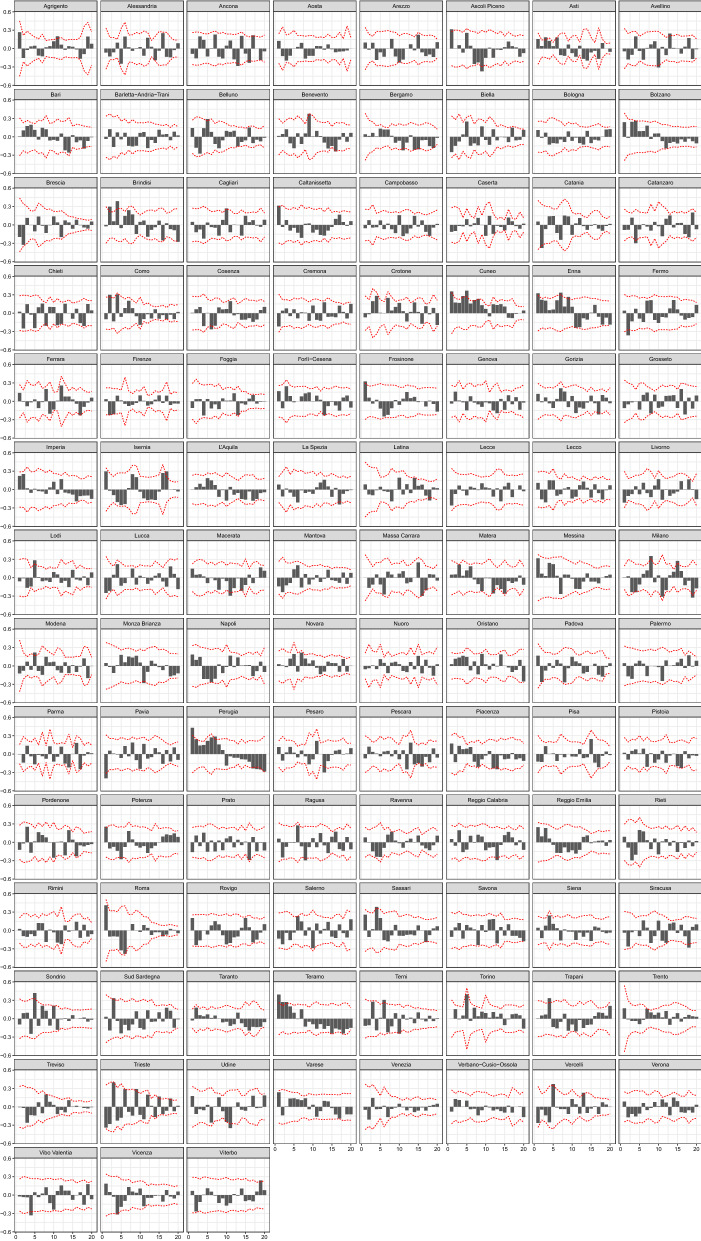
Correlograms of deviance residuals of the fitted model. 95% confidence bands, computed according to [6], are robust with respect to heteroskedasticity

The epidemic-within component—the second term on the right-hand side of Eq. (1)—models the time dependence of the conditional mean $\mu _{r,t}$ through the temporal lag $Y_{r,t-2}$ and the autoregressive parameter $\lambda _{r,t}$. The reason time dependence is modelled through the second order instead of the first-order time lag is twofold. Firstly, temporal autocorrelation of residuals is effectively removed if the second-order lag is included instead of the first-order lag (see Fig. 2). Secondly, the spatial dependence is modelled through the number of contagions of neighbouring provinces at time $t-1$ ($w_{r,h}Y_{h,t-1}$); thus, the use of the second-order time lag (instead of the first order) contributes to the reduction in correlation amongst the epidemic-between and epidemic-within components in Eq. (1).

The temporal autoregressive parameter $\lambda _{r,t}$ determines the contribution of the past number of contagions ($Y_{r,t-2}$) to the current expected number of contagions ($\mu _{r,t}$) within the same region *r*. The parameter $\lambda _{r,t}$ is constrained to be positive and primarily determines the speed of contagions in time. It is thus modelled through a log-linear equation and has the same structure as $\nu _{r,t}$ in Eq. (2):

3 $$\begin{aligned} \ln (\lambda _{r,t})=\alpha _r^{(\lambda )}+\gamma ^{(\lambda )}\,\ln (\text {rpop}_{r}) +\sum _{j=1}^4\beta ^{(\lambda )}_jB_{j,t-t_r}^{(\lambda )}, \end{aligned}$$

where $\alpha _r^{(\lambda )}\sim \mathcal {N}(\alpha ^{(\lambda )},\sigma _\lambda ^2)$ is the random intercept; $\text {rpop}_{r}$ is the relative resident population of province *r*, computed as the ratio between the resident population of province *r* and the average resident population of the Italian provinces, and finally, $B_{j,t-t_r}^{(\lambda )}$ is the value of the *j*th B-spline of the basis computed at time $t-t_r$, $t_r$ being the day when the first contagion in region *r* was detected.

The only remarkable difference between Eqs. (2) and (3) is in the basis of B-splines. In particular, in the case of Eq. (3), the B-splines are computed with respect to time difference from the first contagion in region *r*, that is $t-t_r$. This entails that the basis of B-splines is defined over the interval $[-\max _rt_r\le t\le 56-\min _rt_r]$, and that the evolution of the temporal autoregressive parameter $\lambda _{r,t}$ is homogeneous with respect to the occurrence of COVID-19 in the Italian provinces, which is, as it will be shown in the next section (Fig. 4a), fairly heterogeneous.

The epidemic-between component models the dynamics of contagions between neighbouring provinces by including the average number of infections $Y_{h,t-1}$ recorded the day before ($t-1$) in provinces *h*, which neighbour province *r*. In particular, coefficients $w_{r,h}$ in the summation $\sum _{h\ne r}w_{r,h}\,Y_{h,t-1}$ are positive if provinces *h* and *r* share a border, whereas $w_{r,h}$ are zero otherwise. The coefficient $\phi _{r,t}$ determines the magnitude of the effect of inter-province spread of contagion and changes both in time and amongst provinces.

The spatial autoregressive parameter $\phi _{r,t}$ is modelled following the same approach adopted for $\lambda _{r,t}$, by means of the following log-linear equation:

4 $$\begin{aligned} \ln (\phi _{r,t})= & {} \alpha _r^{(\phi )}+\gamma ^{(\phi )}\,\ln (\text {rpop}_{r})\nonumber \\&+\sum _{j=1}^4\beta ^{(\phi )}_j\,\mathbb {1}(t\ge t_r)\,B_{j,t-t_r}^{(\phi )}, \end{aligned}$$

where $\alpha _r^{(\phi )}\sim \mathcal {N}(\alpha ^{(\phi )},\sigma _\phi ^2)$ is the random intercept; $\text {rpop}_{r}$ is the relative resident population of province *r*; $\mathbb {1}(\cdot )$ is the indicator function; and $B_{j,t-t_r}^{(\phi )}$ is the value of the *j*th B-spline of the basis computed at time $t-t_r$.

Also, in the case of Eq. (4), the basis of B-splines has been adapted. In particular, the basis has been defined over the interval of days after the first contagion detected in each province (that is, $[0\le t\le 56-\min _rt_r]$), whereas the regressors used for including the B-spline functions equal zero for days before the first contagion. This adaptation enables us both to model the post-infection dynamics of parameters (as in the case of $\lambda _{r,t}$) and to reduce the numerical instability of the estimators.

It is worth pointing out that the adaptation of the domains of the basis of the B-splines in Eqs. (3) and (4) is only required because COVID-19 is not endemic to the Italian population; this made it necessary to account for the asynchronous occurrence of the disease amidst Italian provinces.

The estimation of the model has been carried out through the package surveillance [17] implemented in R [21].

### Model assessment

The inclusion of random intercepts in the model equations makes the canonical likelihood-based approaches to significance testing and model goodness-of-fit assessment unfeasible. To deal with this problem, Paul and Held [19] suggested evaluating the performance of the model by assessing its predictive capability with respect to that of alternative competing models using adequate accuracy measures [5]. In this framework, the predictive capability is evaluated through a comparison between the observed values of the time series and the model-predicted ones obtained by sequentially refitting the model up to each day of the time series and computing the one-day-ahead predictions for the corresponding next day [19]. According to Czado et al. [5], a proper comparison between observations and predictions can be made using specific scoring rules for negative binomial predictions that account for uncertainty by considering the predictive distribution instead of the point predictions only. Czado et al. [5] suggest, in particular, using the *logarithmic score* (logs), the *ranked probability score* (rps), the *Dawid–Sebastiani score* (dss) and the *squared error score* (ses). Each has different properties and advantages; therefore, it is advisable to compute all of them in order to evaluate the predictive capability, and hence the goodness of fit, of an estimated model in a comprehensive way [19].

**Fig. 3:**
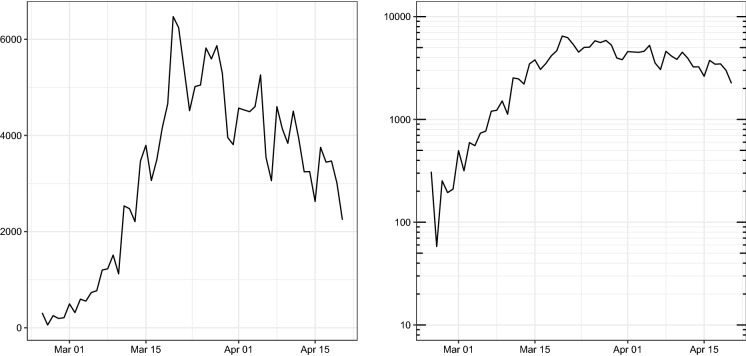
Time series of daily COVID-19 infections in Italy from 24 February 2020 to 20 April 2020, according to data released by the Italian Department of Civil Protection in natural (left) and logarithmic (right) scales. Note the exponential trend of the time series until about 20 March 2020, and the subsequent period when a decreasing trend emerged

The scoring rules herein considered measure, in different ways, the deviation between the fitted model’s predictive distribution, say *P*, and the later observed actual value, say *y*. The *logarithmic score* is given by [11]

$$\begin{aligned} \text{ logs }(P,y)=-\log (P(Y=y)) \end{aligned}$$

This is a proper score which has the characteristic of being “local” since it evaluates the fitted model’s predictive distribution only for the observed value *y* [5]. Due to this, however, it is strongly affected by extreme values as it tends to assign very low scores to counts with low probabilities [19]. A more robust measure in this perspective is the *ranked probability score* ([5, 7]),

$$\begin{aligned} \text{ rps }(P,y)=\sum _{k=0}^\infty (P(Y\le k)-1(y\le k))^2 \end{aligned}$$

which is less sensitive to unusual values as it assigns a relatively higher importance to events with extremely high predicted or observed values [19]. Finally, the more “traditional” *Dawid–Sebastiani score* and *squared error score*, which depend on the fitted model’s predictive distribution only with respect to its moments, are also proper, though not strictly proper, but provide measures that can be interpreted more straightforwardly [10]. They are given, respectively, by

$$\begin{aligned} \text{ dss }(P,y)=\left( \frac{y-\mu _P}{\sigma _P} \right) ^2 +2\log \sigma _P \end{aligned}$$

and

$$\begin{aligned} \text{ ses }(P,y)=(y-\mu _P)^2, \end{aligned}$$

where $\mu _P$ and $\sigma _P$ are the first two moments of the fitted model’s predictive distribution *P* [10].

## Results

### Data

**Fig. 4:**
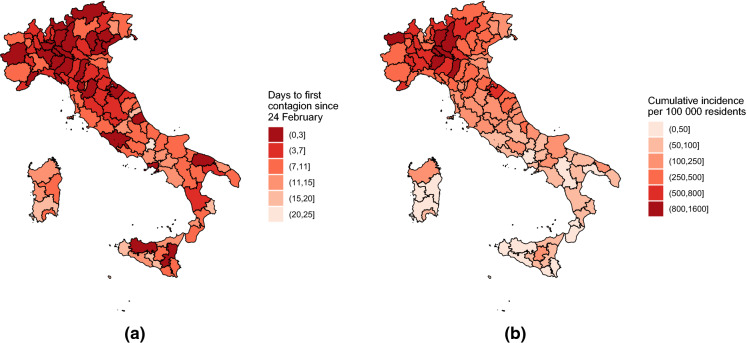
Maps of Italian provinces colour-coded according to the number of days after 24 February, when the first COVID-19 contagion was detected (left), and the cumulative incidence of COVID-19 between 24 February 2020 and 20 April 2020 (right)

The data on COVID-19 infections used to estimate the model are made freely available by the Civil Protection Department of the Italian Government through the official GitHub repository COVID-19 [20]. The repository is updated daily, and possible errors in past collected data are constantly revised. The data refer to the cumulative number of contagions in each of the 107 Italian provinces from 24 February 2020 to 20 April 2020, in which a total of $107\cdot 57=6{,}099$ observations were available. In Fig. 3, the overall number of daily contagions in the country is reported. It is noteworthy that, since the beginning of the epidemic, the number of contagions follows an exponential trend, which starts to decrease around 20 March 2020. Besides data on COVID-19 infections, demographic information about the size of the population of the Italian provinces has also been included in the paper. Those data are freely available on the official website of the Italian Institute of Statistics [14]. Figure 4 presents contagion maps for the 107 Italian provinces in terms of number of days to the first contagion and cumulative incidence, respectively.

**Table 1:** Point estimates and standard errors of parameters of Model (1) based on observations between 24 February 2020 and 20 April 2020

Parameter	Estimate	SE
$\alpha ^{(\nu )}$	2.707	0.151
$\gamma ^{(\nu )}$	1.121	0.161
$\beta _1^{(\nu )}$	$-$ 7.276	0.302
$\beta _2^{(\nu )}$	$-$ 1.665	0.206
$\beta _3^{(\nu )}$	1.130	0.172
$\beta _4^{(\nu )}$	0.429	0.170
$\alpha ^{(\lambda )}$	$-$ 3.732	5.711
$\gamma ^{(\lambda )}$	0.035	0.236
$\beta _1^{(\lambda )}$	9.130	18.111
$\beta _2^{(\lambda )}$	0.462	9.157
$\beta _3^{(\lambda )}$	5.864	4.288
$\beta _4^{(\lambda )}$	$-$ 4.173	10.815
$\alpha ^{(\phi )}$	$-$ 73.125	51.805
$\gamma ^{(\phi )}$	0.596	0.219
$\beta _1^{(\phi )}$	72.228	51.779
$\beta _2^{(\phi )}$	69.835	51.949
$\beta _3^{(\phi )}$	75.259	51.364
$\beta _4^{(\phi )}$	65.374	52.613
$\psi$	0.575	0.015

### Model estimates

Table 1 shows the results for the model described in Sect. 2. The first group of parameters in Table 1 refers to the endemic component $\nu _{r,t}$. Although the parameter estimators are not normally distributed, the large ratios between point estimates and standard errors suggest that all coefficients of the endemic component are highly statistically significant.

**Fig. 5:**
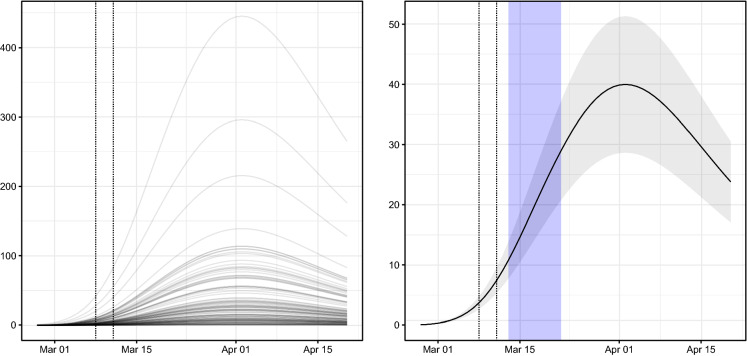
Time evolution of the endemic component $\nu _{r,t}$ of Italian provinces (left) and national average with $95\%$ confidence band (right). Vertical dotted lines mark dates when: (i) the Italian Government issued the RCA; (ii) the Italian Government established the national quarantine. Vertical shaded band highlights the 95% confidence interval of the incubation period of COVID-19 as estimated in [15] for contagions that occurred the day that the DPCM of 11 March 2020 came into force

The coefficient $\gamma ^{(\nu )}$ on province population seems not to be statistically different from 1, suggesting that the contribution of the endemic component of the model is proportional to the population size. On the other hand, intercept $\alpha ^{(\nu )}$ and the coefficients of the B-spline regressors ($\beta _1^{(\nu )}$, $\beta _2^{(\nu )}$, $\beta _3^{(\nu )}$, $\beta _4^{(\nu )}$) are each statistically significant (the Z-scores are larger than 6.5 except for the Z-score of $\beta _4^{(\nu )}$ which equals about 2.5), and the resulting shape of the endemic component is represented in Fig. 5.

As discussed in Sect. 2, the endemic component consists of province-specific effects (the random effect on intercept and the population size) and a common (nationwide) trend. It is worth noting that the inversion of the nationwide trend in Fig. 5 occurs around 1 April, about ten days after the inversion of the overall number of contagions (see Fig. 3). Such a discrepancy should be attributed to the contribution of the dynamics of both the epidemic-within (temporal autoregressive) and epidemic-between (spatial autoregressive) components of the model. This evidence suggests that the local dynamics should not be neglected when studying the diffusion of COVID-19 in Italy.

**Fig. 6:**
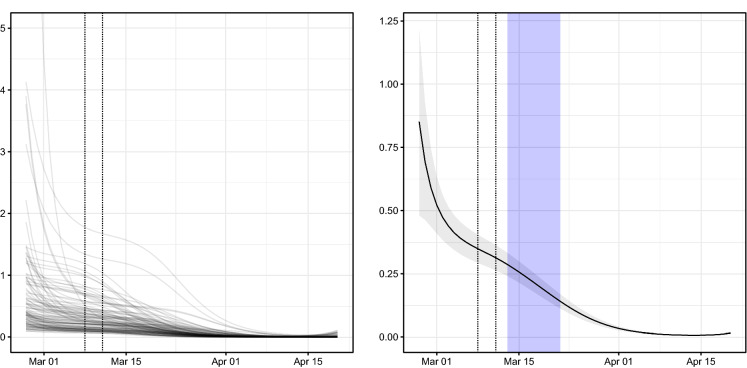
Time evolution of temporal autoregressive parameters ($\lambda _{r,t}$) of the 107 Italian provinces (left) and national average with $95\%$ confidence band (right). Vertical dotted lines mark dates when: (i) the Italian Government issued the RCA; (ii) the Italian Government established the national quarantine. Vertical shaded band highlights the $95\%$ confidence interval of the incubation period of COVID-19 as estimated in [15] for contagions that occurred the day that the DPCM of 11 March 2020 came into force

The second group of coefficients in Table 1 refers to the epidemic-within component, which assesses the strength of intra-province contagion. The evolution in time of this component is represented in Fig. 6, which shows that there is a certain heterogeneity in the value of the temporal autoregressive coefficient ($\lambda _{r,t}$) at the beginning of the outbreak. The large values of $\lambda _{r,t}$ during the first two weeks of the disease diffusion are consistent with the extraordinary speed which characterised the growth in the number of contagions since the very first days when the COVID-19 appeared in Italy. At the end of the first week of March, the value of $\lambda _{r,t}$ was still large for several provinces (see the graph on the left in Fig. 6), whereas a substantial reduction both in the average value and in the proportion of provinces where $\lambda _{r,t}$ was close to or larger than 1 is recorded after 15 March 2020.

The timing of such a change in regime is consistent both with the inversion in the number of overall number of contagions shown in Fig. 3 and with the effects of the government decree issued on 11 March 2020, which extended the quarantine to the whole country. This fact can be checked by looking at the graph on the right in Fig. 6, where a vertical shaded band highlights the 95% confidence interval of the incubation period of SARS-CoV-2 infections that occurred on 11 March 2020, when the government decree came into force. (The duration of the incubation period has been computed according to estimates obtained from [15].) The first effects of the containment measures of the government would be expected to become detectable during that period as long as they were effectively operational. It seems additionally banning the movement of people across towns in the same province had positive effects on the local containment of contagions.

**Fig. 7:**
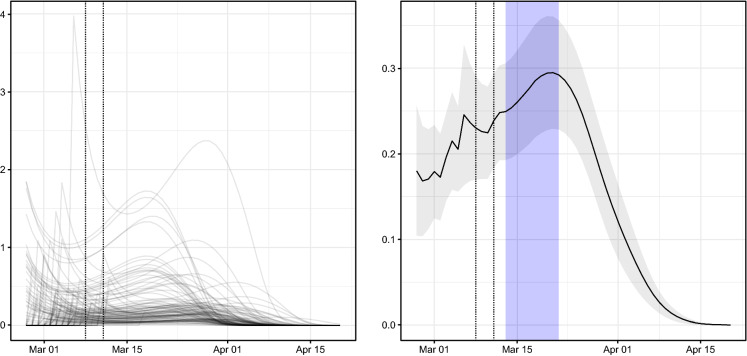
Time evolution of spatial autoregressive parameters ($\phi _{r,t}$) of the 107 Italian provinces (left) and national average with 95% confidence band (right). Vertical dotted lines mark dates when: (i) the Italian Government issued the RCA; (ii) the Italian Government established the national quarantine. Vertical shaded band highlights the 95% confidence interval of the incubation period of COVID-19 as estimated in [15] for contagions that occurred the day that the DPCM of 11 March 2020 came into force

The third group of coefficients in Table 1 refers to the epidemic-between component, which accounts for the contagions amongst neighbouring provinces. Its coefficient $\phi _{r,t}$ is modelled in Eq. (4), and its evolution in time is represented in Fig. 7. As discussed in Sect. 2, coefficient $\phi _{r,t}\ge 0$ allows us to model the spread of contagions amongst neighbouring regions; thus, the larger the value of $\phi _{r,t}$, the larger the contribution of contagions from neighbouring provinces to province *r*. The evolution of the average of $\phi _{r,t}$ consists of two phases. The first period begins when COVID-19 appeared in Italy and concludes around 20 March 2020. During this phase, the average spatial autoregressive parameter grows as COVID-19 spreads amongst Italian provinces at increasing speed and affects the entire territory of Italy (see Fig. 4a). The second phase begins around 20 March 2020 and continues to the end of the period (20 April 2020). During this phase, there are a stabilisation and a rapid decrease in the value of most coefficients $\phi _{r,t}$ (left graph in Fig. 7) and in their average value (right graph in Fig. 7), until they basically reach zero around 15 April 2020. This pattern is consistent with the effects which would be expected from the containment measures of the Italian government, which extended the quarantine to all Italian provinces on 11 March 2020 and stopped, with the government decree DPCM of 22 March 2020, all non-essential businesses and banned any movement inside the country other than for “non-deferrable and proven business or health reasons or other urgent matters” since 25 March 2020 (the so-called *lockdown*).

Finally, it is worth noting that the estimate of the overdispersion parameter $\psi$ in Table 1 justifies the modelling approach based on the negative binomial distribution instead of the Poisson distribution.

**Table 2:** Mean predictive assessment scoring rules based on the last six one-day-ahead predictions for three alternative model specifications

	logs	rps	dss	ses
M1	3.590041	9.750904	6.189101	641.9364
M2	3.605362	10.055681	6.243500	687.5992
M3	3.609414	9.938079	6.207128	697.0025

Lower values indicate better predictions. M1 is the most general model as it includes B-spline regressors in all three components. M2 does not contain B-spline regressors in the within-epidemic component, while M3 excludes them from the between-epidemic part

### Goodness-of-fit assessment

As discussed in Sect. 2, to assess the significance of the estimated model parameters and to evaluate the model goodness of fit, we cannot rely on the standard approaches, which are unfeasible in these circumstances, and we have to resort to focusing on the predictive quality of the estimated model. In particular, following Paul and Held [19], we can conclude that a model has a relatively good fit, and hence, its parameters are globally significant, by whether it leads to better predictions than those provided by other competing models. In this respect, Table 2 compares the prediction ability of our model (labelled M1), which includes B-spline regressors in both the epidemic-within and epidemic-between coefficients, against those of two restricted specifications, namely M2, which excludes the B-spline regressors from the within-epidemic component, and M3, which excludes them from the between-epidemic coefficient. The predictive ability of the three models is assessed by means of proper scoring rules (see Sect. 2.2). Specifically, Table 2 reports the mean scores based on one-day-ahead predictions for the last six days and shows that model M1 has the best predictive performance and hence the relatively highest goodness of fit. Moreover, the results for the *ses* score indicate that the M1 model greatly outperforms the others in terms of mean squared error, implying that it is necessary to include B-spline regressors in all components to obtain an adequate goodness of fit.

## Discussion

**Fig. 8:**
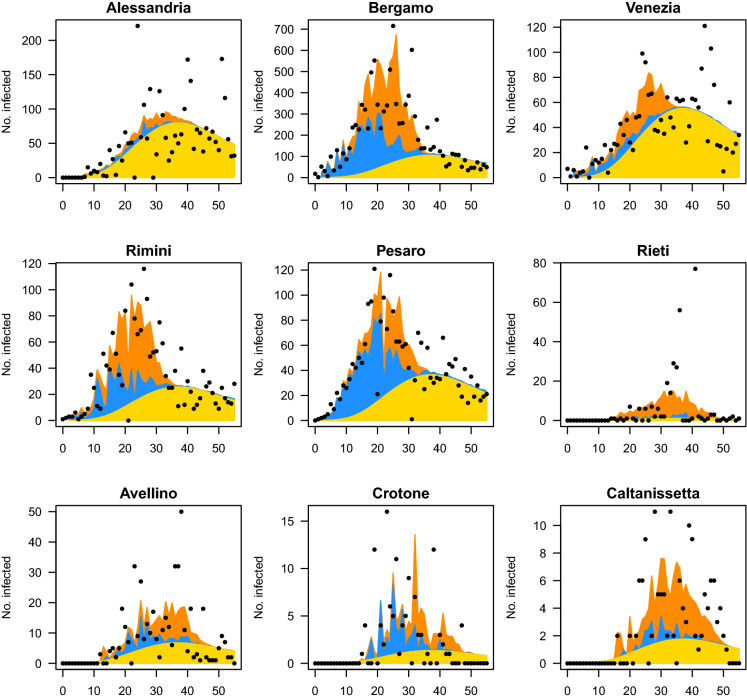
Observed and predicted number of contagions between 24 February 2020 and 20 April 2020 in nine provinces. Selected northern provinces are Alessandria (Piedmont region), Bergamo (Lombardy region), Venezia (Veneto region), Rimini (Emilia-Romagna region) and Pesaro-Urbino (Marche region). The centre and southern provinces are Rieti (Lazio region), Avellino (Campania region), Crotone (Calabria region) and Caltanissetta (Sicily region). The vertical axis represents the daily number of infections, and the horizontal axis represents the time in days after 24 February 2020. The dots represent the observed daily counts. The yellow area represents the endemic component. The light blue area represents the within-epidemic component. The orange area represents the between-epidemic component

The results from the estimated model show that, on average, lockdown measures have succeeded in drastically reducing the transmission of the COVID-19 disease amongst individuals both within and across Italian provinces, as clearly indicated in Figs. 6 and 7. Indeed, the two plots show, respectively, that the estimated temporal and spatial autoregressive parameters started to decrease significantly after the beginning of quarantine. However, the estimates of the random effects and the individual curves of Figs. 6 and 7 also show that the form and extent of the reduction are highly heterogeneous across provinces. To better illustrate this heterogeneity, we examine in detail the cases of nine provinces, five from northern and four from central and southern Italy, which may be considered indicative of various primary trends in the spread of the disease (see Fig. 8).

The first cases of the SARS-CoV-2 virus not directly connected with the Chinese population were diagnosed in certain provinces of Lombardy and Veneto, which then quickly affected other areas in the north of Italy. It seemed that the country was divided into two parts by an imaginary line. Indeed, the epidemic took some weeks to arrive in the central and southern provinces, in some cases, even after the beginning of national quarantine. This occurrence has been previously explained, and it is clearly displayed in Fig. 8, which plots the number of cases estimated by the model along with the actual number of infections. Here, it is also possible to distinguish amongst the three components in order to understand which of these predominate over the others in a province. All of the provinces reported on in the figure exhibit a strong presence of the endemic component (in yellow), the behaviour of which follows the trend of the disease over time. This component reaches its maximum between 35 and 45 days after the beginning of the time series, namely from 19 days after the DPCM of 11 March 2020. More interesting is the behaviour of the temporal (in light blue) and spatial (in orange) autoregressive components, which may provide an idea about intra- and inter-province movements, respectively. If the two components were markedly present in northern provinces since the beginning of the epidemic, they were indeed impeded the moment that containment measures were put into effect. This confirms that blocking movements amongst towns and provinces had effects not only on the global reduction in infections but also on avoiding contagions amongst neighbouring areas. The same cannot be said for the central and southern provinces. We report four emblematic cases which share the circumstance of not having detected any infections at the beginning of the period and the persistence of the two epidemic components even beyond the turning point of the disease trend. These occurrences may be explained only by behavioural patterns of the population: the shared belief that the contagion was not a “local” problem but was restricted to a few distant northern areas might have contributed to sustaining a low perceived risk from COVID-19, which, in turn, reduced the tendency to enact prescribed preventive behaviours, such as distancing and adhering to lockdown rules. Such behavioural patterns of the population in accordance with a low perceived risk result in greater difficulty in stopping the diffusion of the disease, which may continue to bounce around within the area and in neighbouring areas and certainly reduce the effectiveness of the lockdown measures. This shows that the effectiveness of containment measures is influenced by aspects other than biological ones, such as those related to psychological risk perception and citizens’ behaviours under risk and uncertainty [23, 24].

## Conclusion

This paper investigated the impact of lockdown policies in reducing the spread of the COVID-19 disease in Italian provinces. For this purpose, the endemic–epidemic statistical model by Paul and Held [19] was adapted to deal with the complex nonlinear spatio-temporal dynamic of this disease using B-spline regressors. The model’s estimates revealed that on the one hand, containment measures have succeeded in reducing the transmission of infections within and across provinces. On the other hand, however, they also show that the reduction has been highly spatially heterogeneous since the impact of quarantine has been relatively less strong in the provinces where the infections occurred later.

We argue that this heterogeneity can be at least partially explained by psychological and behavioural factors. It is indeed likely that in the provinces where COVID-19 hit earlier and harder, the disease risk perception was higher and instilled greater respect in people for social distancing measures and lockdown rules. Therefore, assessing the relationship between psychographic variables and adherence to quarantine measures may represent an interesting avenue for future research.

On the methodological side, another useful future research direction would be to extend the proposed statistical model to include province-level random effects for the parameters associated with the B-spline regressors. Such an extension would allow better assessment of the heterogeneity across the territory and hence provide further insights into why, in certain provinces, the lockdown has been less effective than in others.
